# Investigating the utility of human melanoma cell lines as tumour models

**DOI:** 10.18632/oncotarget.14443

**Published:** 2017-01-02

**Authors:** Krista Marie Vincent, Lynne-Marie Postovit

**Affiliations:** ^1^ Department of Oncology, Faculty of Medicine and Dentistry, University of Alberta, Edmonton, AB, T6G 2E1, Canada; ^2^ Department of Anatomy and Cell Biology, Faculty of Medicine and Dentistry, University of Western Ontario, London, ON N6A 3K7, Canada

**Keywords:** cell lines, tumour models, microenvironment

## Abstract

Melanoma researchers utilize cell lines to model many tumour phenomena. It is thus important to understand similarities and differences between cell lines and the tumours that they represent, so that the optimal models can be chosen to answer specific research questions. Herein, we compared the transcriptomes of 42 melanoma cell lines to hundreds of tumours from The Cancer Genome Atlas and thousands of single melanoma cells. Tumour purity was accounted for using the ESTIMATE algorithm, so that differences likely resulting from non-tumour cells could be accounted for. In addition, UV mutational signatures and the expression of skin-associated genes were analyzed in order to identify the putative origin of various cell lines. We found the transcriptional and mutational characteristics of melanoma cell lines to mirror those of the tumours, with the exception of immune-associated transcripts, which were absent in cell culture. We also determined cell lines that highly or poorly recapitulate melanomas and have identified colon (COLO 741) and lung (COLO 699) cancer cell lines that may actually be melanoma. In summary, this study represents a comprehensive comparison of melanoma cell lines and tumours that can be used as a guide for researchers when selecting melanoma cell line models.

## INTRODUCTION

Human malignant melanoma is the most deadly form of skin cancer. Although it accounts for only 2% of skin cancer cases, it causes the majority of skin cancer deaths [1]. Furthermore, the incidence of melanoma has been approximately doubling every 10-20 years [2, 3]. While highly treatable if detected early, metastatic melanoma has a five year survival rate of only 10-20% and it remains a particularly aggressive form of cancer [4]. New targeted therapies, such as *BRAF* and immune checkpoint inhibitors, have achieved success in extending patient survival, however, innate or acquired therapy resistance and tumour recurrence is almost unavoidable [5, 6]. Appropriately modeling melanoma is paramount to understanding the molecular mechanisms behind melanoma tumourigenicity and therapy resistance.

Cell lines have been used to model molecular phenomena since the generation of the first immortalized cancer line, HeLa, in 1951. The use of these *in vitro* models has propelled our understanding of molecular cancer biology and led to numerous landmark discoveries, such as the prevalence of *BRAF* V600E mutations in melanoma [7]. However, certain *in vitro* phenomena are often difficult or impossible to replicate *in vivo* and the suitability of cell lines as tumour models has been questioned. Caveats of cell culture include (1) possible selection of a subset of clones particularly amenable to cell culture, (2) loss of *in vivo* microenvironment (eg. three-dimensionality, regions of hypoxia), and (3) loss of stromal, vascular, and immune cellular populations (summarized in [8]). Considering those potential differences, it would not be surprising if cell lines diverged from the tumours they had been established to represent. Understanding the extent to which cell lines accurately represent their parental tumours will help to optimize future research efforts.

Differences in genomic and transcriptional profiles between cancer cell lines and tumour samples have been investigated for several types of cancer including glioma, breast, colorectal and ovarian cancer [9–12]. For example, analysis of high-grade serous ovarian cancer cell lines revealed that the most frequently used cell lines are poorly representative of their tumour counterparts. To aid future research efforts, the authors were able to identify several infrequently used cell lines that more accurately represent their parental tumours in mutational and transcriptional profiles [11]. A comparison of melanoma cell lines to melanoma tumours has been lacking and would likely be of great benefit to the melanoma field.

Recent transcriptional and mutational profiling of 610 cancer cell lines, hundreds of melanoma tumour samples from The Cancer Genome Atlas (TCGA), and thousands of malignant melanoma single cells has enabled a direct comparison of these *in vitro* and *in vivo* groups [13–15]. In this study, we investigate the strengths and weaknesses of cell lines as melanoma models. We show that many melanoma cell lines recapitulate a UV-induced mutational signature; however, there are many transcriptional differences that are primarily driven by clinically relevant immune related genes; thus highlighting the importance of immune system presence in accurate melanoma modeling. In addition, we sought to identify melanoma cell lines that are better models of their tumour counterparts while accounting for *MITF* or *AXL* enriched transcriptional states. Importantly, we show that an annotated lung and an annotated colorectal cell line cluster with and display melanoma characteristics, indicating that they may be of melanoma origin.

## RESULTS

### Comparison of cell lines and tumour expression profiles

**Figure 1:**
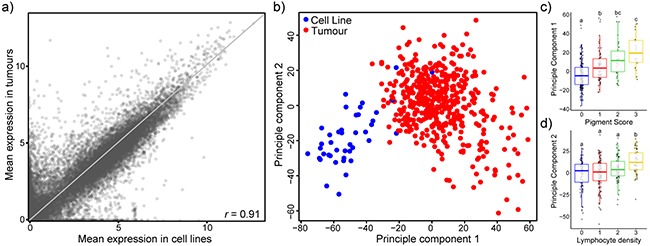
Transcriptional comparison of 42 melanoma cell lines with 471 TCGA melanoma tumour samples suggests overall transcriptional similarity **a**. Scatterplot of mean expression values (log_2_[TPM+1]) of 20,460 coding genes in melanoma cell lines (horizontal) and tumours (vertical). Pearson’s correlation coefficient *r* = 0.91. Grey line depicts the reflection line (y=x). **b**. The 5,000 most variable genes were used for principal component analysis and the first two components are displayed. Most cell lines cluster apart from tumours. Colours of the points indicate sample type: cell line (blue), tumours (red). **c,d**. Boxplots of PC1 broken down by tumour pigment score (c) and PC2 broken down by lymphocyte density (d). ANOVA followed by Tukey’s HSD (p<0.05).

Overall, transcriptional profiles of melanoma cell lines generally resemble that of tumours, with a correlation coefficient of 0.91 for the mean expression of 20,460 coding genes (compared to 0.83 and 0.83 for melanoma tumours correlated to breast and lung cell lines) (Figure 1a, Supplementary Figure 1). However, there are many outliers that have high expression in tumours and little to no expression in cell lines, and *vice versa*. Principal component analysis on expression data of the top 5,000 variable genes reveals two distinct clusters composed mainly of tumours or cell lines (Figure 1b). Interestingly, there are several cell lines that cluster within the tumour group, likely indicative of cell lines that are more transcriptionally representative of tumours. Principal component 1 (PC1) appears to be linked to extent of differentiation, with higher pigment scoring tumours having higher PC1 values (Figure 1c) and cell lines clustering with low pigment scored tumours. This is in line with previous work that has recognized the high representation of amelanotic melanoma cell lines [16]. On the other hand, principal component 2 (PC2) appears to be related to the immune presence, with higher lymphocyte density scoring tumours having higher PC2 values (Figure 1d). Cell lines have comparatively low PC2 values.

### Many of the top differentially expressed genes are immune-related and clinically relevant

**Figure 2:**
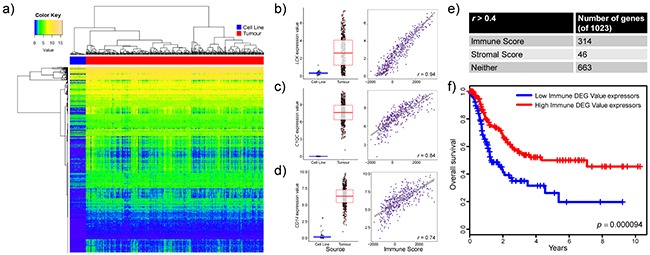
Many of the transcriptional differences between melanoma cell lines and patient tumours are related to immune signatures **a**. Heatmap representation of the top 5% differentially expressed genes (Benjamini-Hochberg adjusted *p* value, Welch’s *t* test) in cell lines compared to tumour samples. All genes were investigated in tumours for potential correlation with the ESTIMATE paradigm stromal and immune scores. **b,c,d**. Differential expression (boxplot) and Immune Score correlations (scatterplot) are displayed for representative genes: (b) *LCK*, (c) *C1QC*, and (d) *CD14*. Boxplots show gene expression values (log_2_[TPM+1]) stratified by sample source (cell line or tumour). Boxes represent interquartile ranges and points represent individual sample values. Scatterplots show gene expression values (log_2_[TPM+1]) versus Immune scores (ESTIMATE algorithm) of tumours. Pearson’s correlation coefficient is displayed in the lower right corner. **e**. Table depicting the number of genes in the top 5% differentially expressed genes that strongly correlate (*r* > 0.4) with the ESTIMATE Immune score, ESTIMATE Stromal score or neither scores. **f**. Kaplan-Meier curves (ten year overall survival) for patients with high Immune DEG Values (red line, n=146) and those with low Immune DEG Values (blue line, n=146). *p* = 0.000094, log-rank test.

To further examine the transcriptional differences between melanoma cell lines and tumours, we focused on the top 5% differentially expressed genes in cell culture (Figure 2a, Supplementary Table 1). Many of the genes found to have high expression in tumours compared to cell lines are known for immune function (eg. *LCK*, *C1QC* and *CD14*; Figure 2b-2c). They were also found to be tightly correlated with tumour-specific Immune scores (as determined by the ESTIMATE algorithm). As well, gene set enrichment analysis of cell lines and tumours revealed lower expression of immune gene sets in culture (eg. hsa04612 Antigen processing and presentation, hsa04650 Natural killer cell mediated cytotoxicity; Supplementary Table 2). To help determine the contribution of immune and stromal cell types to the top differentially expressed genes, gene expression values were correlated to Immune and Stromal scores. 31% (314 of 1023) of the top differentially expressed genes were tightly correlated with Immune score (r > 0.4) while only 4.5% (46 of 1023) were tightly correlated with Stromal score (Figure 2e). Furthermore, the patient-specific summed scaled Z-scores of those 314 immune-related differentially expressed genes (“Immune DEG Values”) were significantly associated with poor overall survival, indicative of the clinical importance of these genes (Figure 2f).

### Ranking of cell lines by transcriptional similarity to tumour counterparts

Tirosh et al. recently conducted single cell RNA-sequencing on over 4,000 stromal and malignant melanoma cells directly extracted from tumours [15]. Using these data we found that cell lines more closely transcriptionally resembled malignant cells than all cells from a tumour (correlation coefficient of mean gene expression of 0.83 and 0.67, respectively). Thus this single cell analysis creates an unprecedented opportunity to directly compare transcriptomes from cell lines (composed entirely of malignant cells) to only malignant melanoma cells directly extracted from tumours. To determine the transcriptional suitability of individual cell lines as tumour models, we ranked the cell lines based on the average correlation coefficient of all genes of all cell line-malignant cell pairs. Although not meant to provide a finely graduated ranking, it can help guide researchers to choose cell lines that are more transcriptionally representative of melanoma patient tumours. This ranking system leads to a spread of melanoma cell lines from most similar to patient tumours (Table 1, *top*) to least similar (Table 1, *bottom*). The top three ranked cell lines (COLO 849, SK-MEL-30, and UACC-257) have high average correlation coefficients of over 0.53, however, they are infrequently used as melanoma models, accounting for only 0.62% of publications on this cell line panel. On the other hand, the bottom three ranked cell lines (Hs 895.T, Hs 852.T, and Hs 839.T) have exceptionally low average correlation coefficients of 0.42, 0.29, and 0.22 and likely represent poor models of melanoma.

**Table 1:** Summary characteristics and ranking of 42 melanoma cell lines based on average Pearson’s correlation of their expression profiles with single malignant melanoma cells

	Mean correlation to						
	all malignant tumour cells	the top MITF program enriched malignant cells	the top AXL program enriched malignant cells	Cell line MITF gene set enrichment	Cell line AXL gene set enrichment	Predominant MITF/AXL cluster	PubMed Citations, n
COLO 849	0.542	0.530	0.508	2.96	-0.39	MITF	0
SK-MEL-30	0.535	0.603	0.458	1.78	-0.28	MITF	15
UACC-257	0.531	0.601	0.451	2.43	-0.80	MITF	18
A375	0.529	0.510	0.512	-3.46	1.32	AXL	1663
537 MEL	0.528	0.521	0.488	0.18	1.54	Intermediate	4
IPC-298	0.523	0.510	0.488	0.25	0.93	Intermediate	6
LOX-IMVI	0.523	0.523	0.490	-3.53	1.96	AXL	39
COLO 792	0.521	0.577	0.447	2.78	-1.99	MITF	0
SK-MEL-28	0.519	0.578	0.459	2.17	-0.64	MITF	451
HMY-1	0.516	0.563	0.466	2.07	-1.91	MITF	4
SK-MEL-2	0.515	0.539	0.475	2.69	-1.48	MITF	250
SK-MEL-31	0.514	0.429	0.524	-0.50	4.30	Intermediate	4
SK-MEL-24	0.514	0.437	0.514	0.46	3.20	Intermediate	32
MeWo	0.513	0.565	0.454	1.81	1.34	MITF	322
HT-144	0.511	0.521	0.476	2.00	0.51	MITF	55
G-361	0.510	0.569	0.459	2.13	-2.76	MITF	287
624-mel	0.510	0.596	0.443	1.15	0.17	MITF	9
MEL-HO	0.507	0.575	0.415	1.75	-1.55	MITF	11
Hs 936.T	0.505	0.554	0.456	2.34	-2.22	MITF	0
SK-MEL-5	0.505	0.579	0.441	1.98	-2.45	MITF	112
IGR-37	0.503	0.566	0.444	1.87	-2.02	MITF	12
MDA-MB-435	0.503	0.474	0.484	-3.33	2.09	AXL	1239
928 mel	0.502	0.570	0.439	2.75	-2.84	MITF	8
COLO 794	0.501	0.457	0.489	-0.84	3.10	Intermediate	0
RPMI-7951	0.500	0.471	0.474	-4.46	3.22	AXL	45
COLO 679	0.500	0.488	0.500	1.14	-1.65	MITF	5
A2058	0.499	0.509	0.458	-1.09	-1.75	MITF	358
C32	0.497	0.434	0.504	-0.88	2.33	Intermediate	157
UCSD-242l	0.488	0.455	0.467	-1.01	3.01	Intermediate	0
SK23	0.484	0.537	0.441	1.88	-0.73	MITF	12
SK-MEL-1	0.483	0.473	0.452	1.84	-1.77	MITF	46
COLO 783	0.482	0.453	0.454	1.04	2.13	Intermediate	0
888 mel	0.473	0.540	0.418	2.15	-2.60	MITF	14
Hs 294.T	0.468	0.440	0.446	-4.33	3.96	AXL	74
SK-MEL-3	0.465	0.532	0.410	2.37	-0.44	MITF	43
Hs 940.T	0.460	0.456	0.431	-3.32	2.35	AXL	0
WM-115	0.449	0.393	0.453	-1.51	3.63	Intermediate	64
Hs 695.T	0.443	0.389	0.441	-1.58	2.69	Intermediate	0
DEOC-1	0.436	0.483	0.383	1.71	1.67	MITF	1
Hs 895.T	0.416	0.370	0.406	-3.18	3.34	AXL	0
Hs 852.T	0.285	0.295	0.248	-2.61	0.46	AXL	0
Hs 839.T	0.216	0.189	0.224	-2.70	2.36	AXL	1

All genes were used to compute the Pearson’s correlation of all the cell line-malignant cell pairs. The cell lines were ranked based on their average correlation indicated malignant cells (n = 1256). MITF and AXL program enriched cells represent the cells with the highest and lowest 400 MITF/AXL enriched cells, respectively. MITF or AXL gene set enrichment is indicated based on GAGE analyses. MITF, AXL or intermediate clusters were structured based on Gaussian kernels.

### Cell lines can be divided by *MITF* or *AXL* transcriptional programs

**Figure 3:**
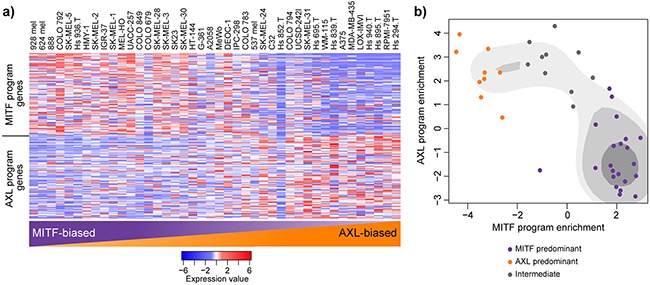
MITF- and AXL-associated expression programs are recapitulated in melanoma cell lines **a**. Relative expression (centered, scaled) of the MITF program genes (top) and AXL program genes (bottom) in 42 melanoma cell lines. Cell lines are sorted based on their gene set enrichment of MITF versus AXL gene sets from highest (left) to lowest (right) as determined by GAGE. **b**. Scatterplot representation of cell line AXL and MITF gene set enrichment as determined by GAGE. Shading represents kernel density estimates of the data. Coloured dots represent clustering as structured by Gaussian kernels (purple: MITF predominant; orange: AXL predominant; grey: intermediate).

In-depth analysis of high-dimensional gene expression data from one cancer type often leads to the identification of discrete and previously unrecognized cancer taxonomy. Bittner et al. were the first group to suggest that there may be transcriptional signatures that define melanoma cell subgroups and further analysis by Hoek et al. defined two main states in melanoma: the proliferative (largely determined by *MITF* expression) and invasive state [17–20]. A recent study of single cell malignant melanoma transcriptomes has further refined these signatures and defined two main transcriptional states of melanoma cells: the *MITF* and *AXL* gene programs [15]. To investigate these newly defined gene programs in melanoma cell lines we investigated the expression of these gene sets in our panel of melanoma cell lines (Figure 3). We found that the cell lines displayed a spectrum of *MITF*/*AXL* gene set expression, but that expression of the two gene sets was often mutually exclusive (Figure 3a). To create *MITF* and *AXL* cell line scores we conducted gene set enrichment analysis of the two *MITF* and *AXL* gene sets. Cluster analysis via kernel density estimation of enrichment values defines three subgroups (a *MITF*-high, *AXL*-high and intermediate group) (Figure 3b, Table 1).

### Ranking of cell lines by transcriptional similarity to malignant cell subgroups

To dissect out some of the subtleties likely due to transcriptional state, we classified the highest and lowest 400 *MITF*/*AXL* ratio single cells as representative of *MITF* and *AXL in vivo* transcriptional states. We then ranked the cell lines based on the average correlation coefficient of all genes of all cell line-*MITF* or *AXL* high malignant cell pairs (Table 1). This analysis revealed cell lines that were highly representative of one transcriptional state but not the other (eg. SK-MEL-30 correlates strongly with *MITF* enriched cells [r = 0.60] but poorly with *AXL* enriched cell [r = 0.46]); or cell lines that were fair transcriptional representations of both states (eg. COLO 849 correlates with intermediate strength to *MITF* enriched cells [r = 0.53] and to *AXL* enriched cells [r = 0.51]). Interestingly, the three cell lines that had the lowest correlations to all malignant cells but that were characterized as *AXL* program enriched, still had low correlations to *AXL*-defined single cell entire transcriptomes, indicating that they are likely poor representations of both *MITF*- and *AXL*-enriched melanoma.

### UV-induced mutational signatures are recapitulated in cell culture

**Figure 4:**
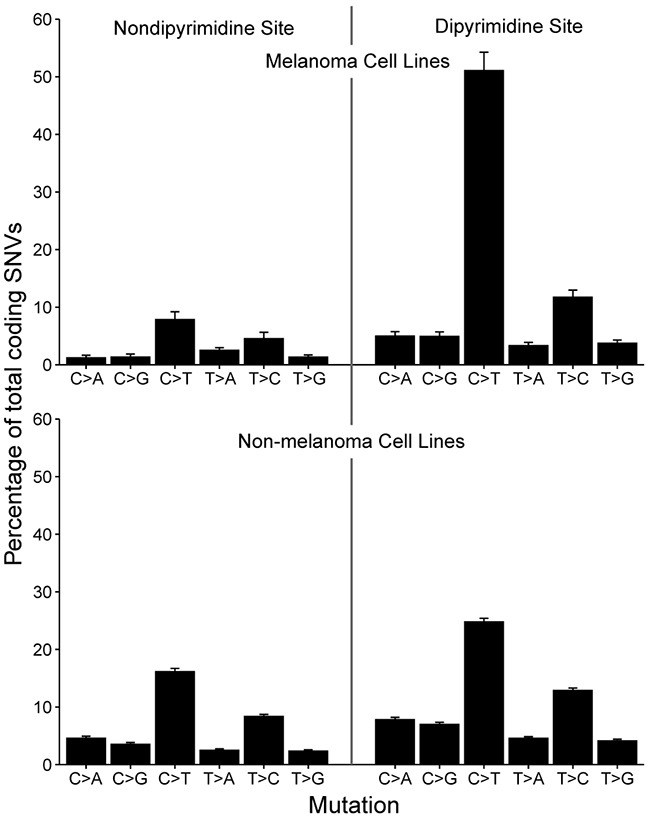
Mutation spectrum in melanoma versus non-melanoma cell lines Barplot depicting the spectrum of somatic variants at dipyrimidine and non-dipyrimidine sites in melanoma (n=42) and non-melanoma (n=567) cell lines. There is an excess of C>T transitions in melanoma cell lines, indicative of UV-exposure and sun-induced DNA damage. Bars denote mean percentage of total SNVs and error bars depict standard error of the mean.

Along with transcriptional identity, another unique feature of over 75% of patient melanoma samples is the presence of a UV-induced mutational signature [14]. UV signature mutations are defined by an enrichment of C>T substitutions at dipyrimidine sites. To see if this phenomenon was replicated *in vitro*, we assessed the mutational spectrum of annotated melanoma versus non-melanoma cell lines and found that the average melanoma cell lines had over a two-fold increase in C>T substitutions at dipyrimidine sites (Figure 4). This indicates that melanoma cell lines can maintain a UV-induced mutational signature in culture, consistent with prior studies [21, 22].

### Tissue of origin analysis reveals potential mis-identified melanoma cell lines

**Figure 5:**
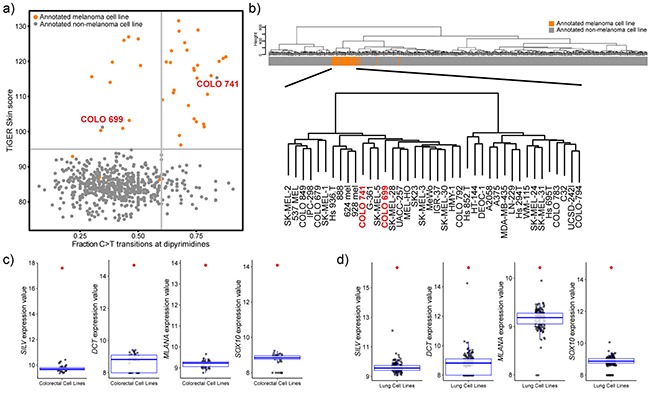
Tissue of origin analysis for melanoma cell lines **a**. Scatterplot representation of fraction of C>T transitions at dipyrimidine sites and the weighted expression of skin genes. Vertical line at x=0.6 delineates UV mutational signature positive (>0.6) from UV mutational signature negative samples (<0.6). Horizontal line depicts a TiGER Skin Score of 95 and delineates the majority of melanoma samples from non-melanoma samples. **b**. Unsupervised hierarchal clustering of 610 cell lines based on gene expression data (VSD normalized values) of the 5,000 most variable genes. Clustering was done using Euclidean distance and Ward linkage. The bar represents the cell line tissue of origin (orange: melanoma cell line; grey: non-melanoma cell line). Bottom clustering depicts a closer view of the melanoma cluster and shows that COLO 741 and COLO 699 cell lines cluster with melanoma samples. **c,d**. Boxplot representation of the expression (VSD normalized values) of skin-specific genes: *SILV*, *DCT*, *MLANA*, and *SOX10* in annotated colorectal cell lines (c) and annotated lung cell lines (d). Red points represent the values for the COLO 741 cell line (c) or the COLO 699 cell line (d).

Another unique feature of melanoma is its aggressive nature—melanomas have the ability to metastasize when the primary tumour thickness is less than 1 mm [23]. Furthermore, no primary lesion can be identified in 13-17% of patients presenting with palpable evidence of regional metastatic melanoma [24]. This, along with the potential for cell lines to become cross-contaminated or misidentified at any point during their extensive culture history, leads to greater than normal potential for melanoma cell lines to be misidentified as other types of cancer. To investigate this possibility in this cell panel, TiGER (tissue-specific gene expression database) was utilized to perform a skin-specific tissue of origin analysis along with classification based on the UV-induced mutational signature (Figure 5a). The analysis revealed a probable melanoma-origin for two annotated non-melanoma cell lines: COLO 741 (annotated colorectal origin) and COLO 699 (annotated lung origin). Along with this, in unsupervised hierarchal clustering, both the COLO 741 and COLO 699 cell lines cluster in the melanoma-specific branch (Figure 5b) and express skin-specific genes (eg. *SILV*, *MLANA*, *DCT* and *SOX10*) at such high levels that they are outliers when compared to all other annotated colorectal (Figure 5c) or lung cell lines (Figure 5d). On the other hand, the transcriptionally least representative melanoma cell lines (Hs 895.T, Hs 852.T, and Hs 839.T; Table 1) do not cluster with melanoma samples in TiGER skin-specific analysis or in unsupervised hierarchal clustering.

### Genomic characterization of melanoma cell lines

**Figure 6:**
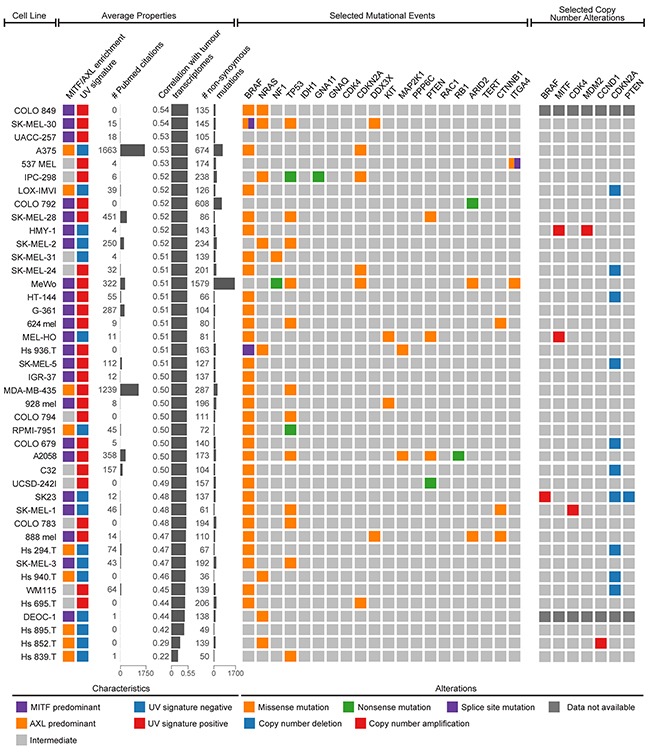
Genomic summary of melanoma cell lines Both average properties (left) and selected genetic events (right) can be used to choose the optimal cell model for specific occasions. Average properties include MITF/AXL/intermediate clustering, UV signature status, the citation frequency in the literature, the average transcriptional correlation with malignant melanoma cells, and the number of non-synonymous mutations. The selected genetic events include 20 possible somatic mutations (*BRAF*, *NRAS*, *NF1*, *TP53*, *IDH1*, *GNA11*, *GNAQ*, *CDK4*, *CDKN2A*, *DDX3X*, *KIT*, *MAP2K1*, *PPP6C*, *PTEN*, *RAC1*, *RB1*, *ARID2*, *TERT*, *CTNNB1*, and *ITGA4*) and 7 possible copy number alterations (*BRAF*, *MITF*, *CDK4*, *MDM2*, *CCND1*, *CDKN2A*, and *PTEN*) determined to be significantly altered in the landmark melanoma study by The Cancer Genome Atlas.

To summarize transcriptional analyses and mutational and copy number alterations observed in melanoma cell lines, we assembled a tabular resource, which can be used by researchers when choosing cell line models (Figure 6, Supplementary Table 3). In general, the frequencies of these somatic mutational events in melanoma patient samples are replicated in melanoma cell lines with two exceptions: *BRAF* and *TP53* are mutated at significantly higher frequencies in cell lines (p<0.05 by binomial test; Supplementary Table 4).

## DISCUSSION

This is the first study that employs next generation sequencing data to investigate the suitability of melanoma cell lines as tumour models. We have demonstrated that in general, melanoma cell lines recapitulate the mutational and transcriptional profiles of patient tumours with a notable exception: immune profiles of patient tumours are absent in cell lines. Therefore, the clinically important immune landscape of melanoma is poorly represented *in vitro* and alternative tumour models should be considered to more appropriately represent these scenarios. In addition, we were able to rank cell lines based on their transcriptional similarity to tumours—recommending that cell line choices be informed by this summary. We have also pointed out annotated colorectal and lung cell lines that are of probable melanoma origin, and identified several annotated cell lines that are exceptionally poor models of melanoma. We anticipate that this analysis will help the melanoma field make more informed choices regarding model selection for each specific research question.

In general, we found that melanoma cell lines were transcriptionally representative of their respective tumours, with the majority of genes having a similar mean expression in cell lines and tumours. However, there were a subset of genes with drastic differences in expression levels, predominantly in the direction of high expression in tumours and little to no expression in cell lines. When we investigated the identity of these top differentially expressed genes, we found that many of them are known immune genes and tightly correlated with immune presence. This is not surprising given the immunogenic nature of melanoma and the absence of immune cells in cancer cell monoculture. Melanoma cell monoculture fails to address the role of the immune system. Hence, phenotypic behaviors observed *in vitro* may not always translate due to unforeseen interactions with immune cells *in situ*, and without syngeneic models, significant biological consequences of affecting immunogenicity could be missed.

Fortunately, there are murine models of melanoma, including the popular B16 cell line series, which may allow the melanoma field to overcome some of these difficulties. The B16 series of cell lines are derived from a spontaneous melanoma in C57BL/6 mice [25]. Thus the cells can be implanted back in this murine strain within the context of an intact immune system as a syngeneic model of melanoma [26]. However, it should be recognized that animals provide only an approximation of the reality in humans. Due to differences in skin architecture, mouse melanomagenesis does not exactly phenocopy disease progression in humans (reviewed in [27]). Mice, as heavily hair-covered species, have no need for skin pigmentation, and therefore almost completely lack epidermal melanocytes. Instead, melanocytes are located in dermal hair papilla. Thus, unlike most human melanomas that are of epidermal origin and usually lack pigment, most murine lesions are dermal with very high levels of pigment. In addition, the most commonly used murine melanoma model (B16) does not harbor a *BRAF* mutation [28]. Accordingly, murine melanomas likely do not recapitulate some of the key features of human melanoma and come with a different set of caveats.

Despite the transcriptional differences that we observed between human melanoma cell lines and tumours, we did observe cell lines that clustered closer to their tumour counterparts than others in the principal component analysis. Accordingly, we predicted that some cell lines would more accurately represent the transcriptional profile of melanoma tumours. Recent single cell RNA-sequencing of over 4,000 melanoma cells provides an unprecedented opportunity to directly compare the trancriptomes of *in vivo* malignant melanoma cells with cell lines (composed entirely of malignant cells). Our analysis found that the correlation coefficient of individual cell lines to malignant cells varied from 0.22 to 0.54. This was somewhat similar to the ranges of 0.41-0.58 and 0.43-0.60 that has been previously observed in similar analyses conducted on breast and ovarian cell lines, respectively [10, 11]. It also revealed instances wherein certain cell lines better represented one transcriptional state over another (eg. SK-MEL-30 cells have a high average correlation coefficient to *MITF*-defined malignant cells [r = 0.60] but not *AXL*-defined malignant cells [r = 0.46]). While several melanoma cell lines appear to be good tumour models, there are also three cell lines (Hs 895.T, Hs 852.T, and Hs 839.T) that display exceptionally poor transcriptional resemblance to their tumour counterparts. As well, these three cell lines cluster with non-melanoma cell lines using tissue of origin analyses and unsupervised hierarchal clustering. While it’s difficult to conclude if these three cell lines have been misidentified as melanoma lines, it is convincing that they represent exceptionally poor models of melanoma.

On the other hand, it may be more feasible to identify annotated non-melanoma cell lines as being of probable melanoma origin. The precedent for this was set in 2000, when the annotated breast cancer MDA-MB-435 cell line was shown to cluster with melanoma cell lines and express melanoma-specific genes [29]. Follow-up analysis revealed the likelihood that all available MDA-MB-435 cells are derived from the M14 melanoma cell line [30]. The decades of extensive breast cancer-specific literature conducted on this cell line represent a valuable resource for the melanoma field. In our tissue of origin analysis and unsupervised hierarchal clustering, we identified two annotated non-melanoma cell lines that are probably of melanoma origin: the COLO 741 annotated colorectal cell line and the COLO 699 annotated lung cell line. A separate study by Medico et al. of 151 colorectal cell lines has previously questioned the origin of COLO 741 cells [12]. It is difficult to tell if these probable misidentifications are a result of cell line cross-contamination, like in the case of MDA-MB-435 cells, or a result of an original incorrect tumour diagnosis. However, we can conclude through the SNP profiling conducted by Klijn et al. on 675 cell lines, that while the COLO 741 cell line was genetically dissimilar to all other cell lines tested, the COLO 699 cell line was highly concordant to the annotated melanoma cell line CHL-1 and may be a result of cross-contamination. Until a thorough investigation into the background of these two cell lines is conducted, we recommend that researchers exercise caution when interpreting results obtained using them.

This study is the first of its kind to determine the extent to which melanoma cell lines represent tumours in terms of gene expression and mutational burden. While generally quite similar, we determined that cell lines and tumours differ at the transcriptional level, likely due to a loss of immune components in cell culture. Additionally, we were able to rank cell lines based on their transcriptional similarity to malignant cell tumour counterparts and identified three cell lines that were exceptionally poor models of melanoma. We have also identified two cell lines, COLO 741 and COLO 699, which are of probable melanoma origin. Knowing the strengths and weaknesses of our widely used tumour models can help direct tumour model choice and improve the clinical value of future research efforts.

## MATERIALS AND METHODS

### Datasets

Level 3 TCGA RNAseqV2 gene expression and Level 2 TCGA somatic mutation data (BI mutation calling) was obtained from the TCGA Data Portal in January 2016. Patient survival information, pigment scores and lymphocyte density values for TCGA samples was obtained from the supplementary information of the SKCM TCGA landmark paper (original publication [14]). RNA-sequencing expression, mutational and copy number information was retrieved in January 2016 for 675 cancer cell lines from the supplementary information of Klijn et al. (original publication [13]). Cell line analysis and details, including growth conditions and sequencing methods, are described in the original publication. Single cell RNA-sequencing of malignant melanoma cells were obtained from Gene Expression Omnibus GSE72056 in June 2016.

### Data preparation

Relative abundance (transcripts per million, TPM) was calculated for 471 melanoma patient samples (104 primary tumours and 367 metastases) by multiplying the scaled estimate data by 10^6^, and for 42 melanoma cell lines by converting RPKM (reads per kilobase of exons per million mapped reads) to TPM. To avoid infinite values in log calculations, a value of 1 was added to TPM values before log_2_ transformation. Values for the genes that were available on both datasets were used in downstream analyses (20,460 coding genes in total). For single cell analysis, TPM values were divided by 10 as in the original paper, given that the complexity of single cell libraries is estimated to be in the order of 100,000 transcripts.

### Gene expression profiling analysis

The top 5,000 genes by variance across the combined cell line-tumour dataset were chosen for principal component analysis. Significant differences in relative transcript abundances between cell lines and tumours were calculated with Welch’s t test and p values were corrected for multiple testing using the Benjamini-Hochberg method. Unsupervised hierarchal clustering was done using Euclidean distance and Ward’s agglomeration method (ward.D2). Enrichment for functionally related genes between the two datasets was tested using Generally Applicable Gene-set Enrichment (GAGE, v2.12.3) with KEGG gene sets.

### Tumour purity

Stromal and Immune scores were defined for tumours through the use of the ESTIMATE (Estimation of STromal and Immune cells in MAlignant Tumour tissues using Expression data; original publication [31]) algorithm using RNASeqV2 data. Pearson’s correlation coefficient was used to calculate the association of specific genes to Stromal and Immune scores.

### Survival analysis

Immune Differentially Expressed Gene (DEG) Values were calculated for tumours by creating and summing Z-scores for genes that were (1) in the top 5% of differentially expressed between cell lines and tumours and (2) strongly correlated with immune score (r > 0.4). Immune DEG Values were dichotimized by median and ten year survival curves were constructed using the Kaplan-Meier method on metastatic samples that had survival information available (n=292). Significance was determined by log-rank test.

### *MITF*/*AXL* program analysis

*MITF* and *AXL* gene programs as defined by Tirosh et al. were used to characterize cell lines [15]. Enrichment for gene sets in the cell lines was tested using Generally Applicable Gene-set Enrichment (GAGE, v2.12.3) with Tirosh et al. defined gene sets. Kernel density estimation was conducted on *MITF* and *AXL* cell line enrichment scores and clusters were defined using Gaussian kernels.

To define the top *MITF* and *AXL* gene set enriched single melanoma cells, *MITF* and *AXL* cell scores were calculated as in the original paper [15] and a ratio of the two values were taken to rank the malignant cells. Briefly, we defined both *MITF*/*AXL* average relative expression cell scores and control gene set cell scores. Control gene set scores were subtracted from their respective *MITF*/*AXL* cell scores. All genes were used to compute Pearson’s correlation of all cell line-single malignant cell pairs and cell lines were ranked based on their average correlation with all malignant cells. In *MITF*/*AXL* specific analyses, Pearson’s correlation of the malignant single cells with the highest or lowest 400 *MITF*/*AXL* ratios were used instead of all malignant cells.

### Mutational analysis

Non-synonymous coding mutations with neighbouring nucleotide information were extracted from all non-synonymous coding mutations and used in mutational spectrum analyses. Patterns of particular nucleotide substitutions were determined for melanoma (n=42) and non-melanoma (n=567) cell lines as a mean percentage of total somatic variants. Cell lines were classified as UV signature positive if the fraction of C>T transitions at dipyrimidines was >0.6 as defined in the landmark TCGA SKCM paper [14].

In our genomics summary, selected mutational events and copy number alterations were considered if they were found to be significantly altered from normal tissue in the landmark TCGA SKCM study [14]. Copy number alterations represent ploidy-corrected copy number calls; copy number amplifications represent ploidy-corrected copy number values > 1 and copy number deletions represent ploidy-corrected copy number values < -0.75.

### Tissue of origin analysis

The skin tissue-specific panel of genes was retrieved from the TiGER portal (bioinfo.wilmer.jhu.edu/tiger/). Gene symbols were filtered for presence in the SKCM RNA-sequencing dataset. TiGER Skin Scores were determined for each cell line as previously described [12]. The TiGER database provides an “EST Enrichment” (EE) score, proportional to enrichment in that specific tissue. To weigh the genes more strongly associated with skin-specific expression, EE scores were squared and summed, and the squared EE square of each gene was divided by the sum to obtain a scaled EE score. The cell line specific TiGER Skin Scores were calculated by summing the products of gene expression (VSD normalized values) and the scaled EE score.

### PubMed citation analysis

The number of PubMed abstracts that mentioned one of the 42 melanoma cell lines was determined as an estimator of frequency of use. Hits were determined using the PubMed search function (www.pubmed.com) on February 29, 2016. Several punctuation alternatives were used for the cell line names.

### Statistical analysis

We conducted all analyses and visualizations in the RStudio programming environment (v0.98.501). R/Bioconductor packages ggplot2, plyr, gplots, ggdendro, survival, pdfCluster, and GAGE were used where appropriate.

## SUPPLEMENTARY MATERIALS FIGURES AND TABLES









## Abbreviations

TCGA: The Cancer Genome Atlas
TPM: transcripts per million
RPKM: reads per kilobase of transcript per million reads mapped
ESTIMATE: Estimation of Stromal and Immune cells in Malignant Tumour tissues using Expression data
GAGE: Generally Applicable Gene-set Enrichment
SKCM: skin cutaneous melanoma
DEG: differentially expressed gene
EE: EST Enrichment
PC1: principal component 1
PC2: principal component 2
