# Supplementary material for: Investigating the utility of human melanoma cell lines as tumour models
Source: Oncotarget. 2017 Jan 2;8(6):10498–509. doi: 10.18632/oncotarget.14443 (PMC5354675; doi:10.18632/oncotarget.14443)
Supplement: Supplementary file 1 [file oncotarget-08-10498-s001.pdf]

Investigating the utility of human melanoma cell lines as tumour models

SUPPLEMENTARY FIGURE AND TABLES

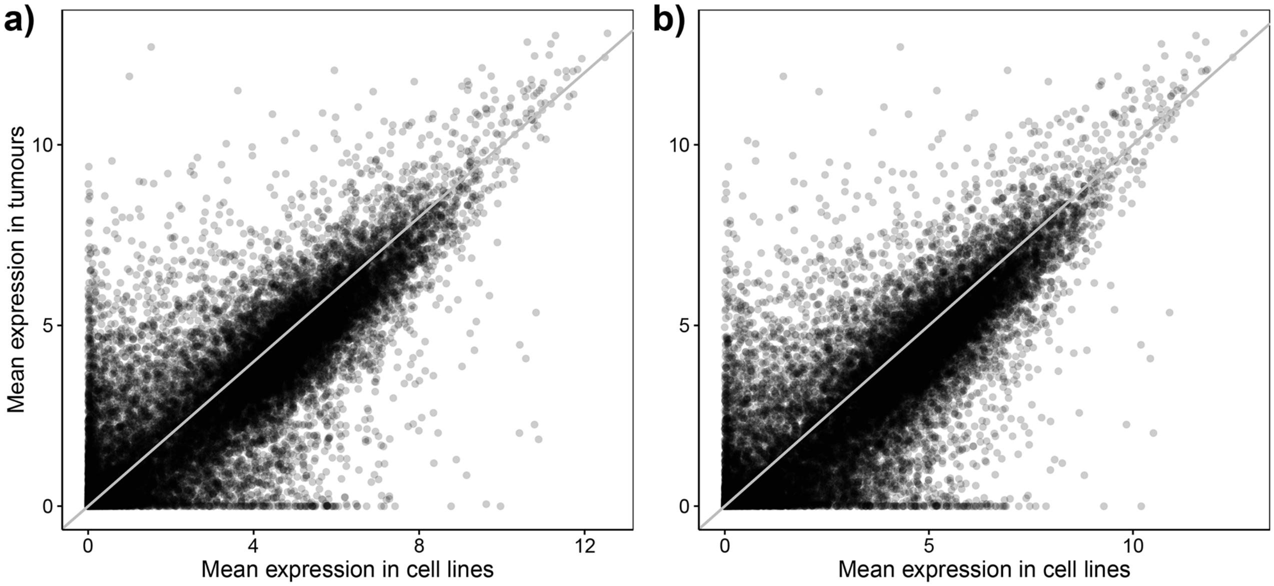

Supplementary Figure 1: Transcriptional comparison of 471 TCGA melanoma tumour samples with a. 61 breast cancer cell lines and **b.** 144 lung cancer cell lines. Scatterplot of mean expression values (log_2[TPM+1]) of 20,460 coding genes in cell lines (horizontal) and tumours (vertical). Pearson's correlation coefficient r = (a) 0.83 and (b) 0.83. Grey line depicts the reflection line (y=x).

Supplementary Table 1: Top 5% of differentially expressed genes between melanoma cell lines and tumour samples.

See Supplementary File 1

Supplementary Table 2: Top five upregulated and downregulated KEGG gene sets by Generally Applicable Gene set Enrichment analysis (GAGE) between melanoma cell lines and tumour samples

**Upregulated Sets**		
**KEGG Gene Set**	**Mean t-statistic**	**Set Size**
hsa04110 Cell cycle	1.54	122
hsa04141 Protein processing in the endoplasmic reticulum	1.26	161
hsa03040 Spliceosome	1.26	124
hsa03030 DNA replication	1.11	36
hsa04114 Oocyte meiosis	1.07	111
**Downregulated Sets**		
**KEGG Gene Set**	**Mean t-statistic**	**Set Size**
hsa03010 Ribosome	-5.86	88
hsa04612 Antigen processing and presentation	-1.01	69
hsa04514 Cell adhesion molecules (CAMs)	-0.99	130
hsa04650 Natural killer cell mediated cytotoxicity	-0.94	131
hsa00860 Porphyrin and chlorophyll metabolism	-0.93	41

Supplementary Table 3: Specific mutations of driver genes in melanoma cell lines.

See Supplementary File 2

Supplementary Table 4: Comparison of the frequency of select mutational events in melanoma cell lines *versus* tumours. Differences in mutational frequencies of selected genes in and melanoma cell lines (*n* = 42) and TCGA melanoma tumours (*n* = 294).

See Supplementary File 3
